# Acute toxicity of commercial atrazine in *Piaractus mesopotamicus*: Histopathological, ultrastructural, molecular, and genotoxic evaluation

**DOI:** 10.14202/vetworld.2017.1008-1019

**Published:** 2017-09-01

**Authors:** Paula Pereira de Paiva, Mariana Cruz Delcorso, Valquíria Aparecida Matheus, Sonia Claudia do Nascimento de Queiroz, Carla Beatriz Collares-Buzato, Sarah Arana

**Affiliations:** 1 Department of Biochemistry and Tissue Biology, University of Campinas (UNICAMP), Po. Box 6109, 13083-970, Campinas, SP, Brazil; 2 Laboratory of Residues and Contaminants, Embrapa Environment, Jaguariúna, SP, Brazil

**Keywords:** Brazilian ichthyofauna, kidney lesions, liver lesions, micronucleus test, oxidative stress

## Abstract

**Aim:**

The aim of this work was to evaluate the sensitivity of Pacu fingerlings (*Piaractus mesopotamicus*) by measuring the effects of median lethal concentration (LC_50_) of atrazine (ATZ - 28.58 mg/L) after acute exposure (up to 96 h).

**Materials and Methods:**

The fish were exposed to the LC_50_ of ATZ for 96 h (28.58 mg/L) in a static system. During the experiment, the fingerlings were randomly distributed in four glass tanks (50 L) containing dechlorinated water. Four glass tanks were for the control group, and four were for the ATZ-exposed group (n=4 per glass tank), given a total number of 16 animals tested per group. The genotoxicity was evaluated by micronucleus (MN) test in erythrocytes from peripheral blood. Qualitative and semi-quantitative histopathological analyses, and also ultrastructural study, were applied in liver and kidney samples. Finally, the content of heat shock protein (Hsp70) in the liver was evaluated by the western blotting method.

**Results:**

The morphological alterations in the liver, which was associated with increased expression of Hsp70, included nuclear and cytoplasmic vacuolization, cytoplasmic hyaline inclusions, and necrosis. The kidney presented edema and tubular cell degeneration with cytoplasmic hyaline inclusion. The semi-quantitative histopathological analyses indicated that the liver was more sensitive than kidney to ATZ-induced damage. Ultrastructural analysis showed that ATZ caused membrane alterations in several organelles and increased the number of lysosomes in hepatocytes and kidney proximal tubular cells. Nevertheless, no significant difference was observed in MN frequency in erythrocytes comparing treated and control groups.,

**Conclusion:**

These results indicated that ATZ-induced damage to the kidney and liver function, ATZ at the concentration tested did not induce a significant difference in MN frequency in Pacu erythrocytes comparing treated and control groups, and also that Pacu fingerlings may be a good bioindicator for testing freshwater contamination.

## Introduction

The intense use of atrazine (ATZ) and its environmental persistence both in soil and the aquatic environment are a matter of great concern because of the environmental impact it may cause. Indeed, taking into consideration these risks not only to the environment but also to human health, ATZ was banned by the European community [1]. Nevertheless, ATZ is widely used in sugarcane, corn plantations, and other agricultural crops in several countries, such as the United States [2] and South America, including Brazil [3].

Particularly in Brazilian rivers, the presence of ATZ has often registered at permitted levels (2 µg/L) established by the Brazilian National Council of the Environment (CONAMA) [4-7]. However, ATZ was found above this level in several hydrographic basins in different regions of Brazil, ranging from a concentration of 2.7 µg/L [8], 10.4 µg/L [9], and 15.0 µg/L [10] up to 18.9 µg/L [11].

Despite the high frequency of reports of the presence of ATZ in Brazilian river basins, few studies have examined the sensitivity of the Brazilian ichthyofauna to this compound [9,12-15].

In fish species from temperate regions, environmentally relevant concentrations of ATZ have been associated with many harmful effects. Histopathological analysis indicated that ATZ causes the induction of many extensive hepatic and renal lesions [16-20]. These two organs are directly related to the detoxification process and are indicators of systemic toxicity induced by xenobiotics present in the aquatic environment, but similar histopathological studies have rarely been reported in neotropical fish species exposed to ATZ [9,21-23]. The relationship between ATZ exposure and cellular stress induction has also been shown in fish, employing biochemical analysis [24,25], including Neotropical species [26]. However, few works have specifically evaluated the expression of heat shock proteins (Hsps) in fish exposed to ATZ [27,28], despite the fact that this family of proteins has been widely applied in biomonitoring and ecotoxicology because of their responsiveness to diverse forms of stress [29]. However, there is no study investigating this protein in Brazilian Teleosts exposed to this herbicide.

In addition, a controversial aspect related to the ATZ toxicity has been its genotoxic and mutagenic potential. Several studies in mammals and fish have indicated that only the commercial formulation of ATZ causes genotoxic effects, suggesting that some adjuvant in the commercial formulation accounts for this toxic property [30,31]. In Brazilian icthyofauna, as far as we know, the genotoxicity of commercial ATZ has never been investigated.

Considering what is known about ATZ, its toxic effects and its potential risks to Brazilian ichthyofauna, this work aimed to investigate the sensitivity of Pacu fingerlings (*Piaractus mesopotamicus*) to this herbicide. This fish species was chosen based on its ecological and economic importance and its wide geographical distribution in Brazilian river basins. An acute toxicity assay (up to 96 h) using the median lethal concentration (LC_50_) of a commercial formulation of ATZ determined in Pacu was performed. The following parameters were analyzed as follows: (1) The histopathological, ultrastructural aspects of the liver and kidney; (2) the level of hepatic expression of Hsp70, used as an index of cellular stress, which was evaluated by the western blotting; and (3) the genotoxic potential, which was determined in erythrocytes from peripheral blood using a micronucleus (MN) assay.

## Materials and Methods

### Fish maintenance

Pacu fingerlings (*P. mesopotamicus* - Holmberg 1887) were obtained from a fish farm located in Mogi Mirim in São Paulo state (−22° 25’ 55’’ S and −46° 57’ 28’’ W).

The fingerlings were transferred to stock tanks in the laboratory containing dechlorinated and aerated tap water, where they were kept for 30 days for acclimation. The water quality was monitored daily for the pH value (QUIMIS 186, 400A model), dissolved oxygen levels (oximeter – QUIMIS, Q-758P model), toxic ammonia concentration, and once a week for the total hardness, the last parameters were evaluated using colorimetric kits (Kit Labcon test – Alcon^®^). The photoperiod was established as a 12/12 h light/dark cycle controlled by a timer. The fish were fed daily with a commercial fish feed containing 4-6 mm pellets (Pirá 36, Guabi Nutrição Animal) at a rate of 1.5% of their mean body weight. On alternate days, approximately one-third of the volume of the water in the tanks was siphoned for the removal of the remains of organic matter, and this water was immediately replaced, maintaining the same initial characteristics.

Before the experiment, the fish were maintained for 7 days for acclimatization to the glass aquaria at similar conditions to those of the stock tank, i.e., water pH 8.10±0.08, temperature 21.86±0.08°C, dissolved oxygen 8.61±0.46 mg/L, ammonia concentration 0.017±0.005 ppm, and hardness bland. The diet was suspended 2 days before the beginning of the toxicity assays.

### Chemicals

A solution was prepared with the commercial herbicide ATZ purchased from the market (6-chloro-N2-ethyl-N4-isopropyl-1,3,5-triazine-2,4-diamine; 500 g/L Gesaprim 500; CIBA-GEIGY Syngenta - 50% m/v active ingredient). The herbicide was dissolved in distilled water to obtain a stock solution. From this solution, the required amount was used to obtain a nominal concentration of 28.58 mg/L for each glass aquarium. The anesthetic 2-phenoxyethanol was purchased from Sigma (St. Louis, MO, USA), and the EMbed 812 epoxy resin – Epon kit was purchased from EMS (Hatfield, PA, USA). All other chemicals used were analytical grade.

### Analytical chemistry of the water sampling

To determine the concentration of ATZ in the water from the tanks during the experiments, 100 mL of water was collected from the treated and control groups 1 h, 24 h, and 96 h after the addition of test solutions to the aquaria. The samples were transported and kept under refrigeration until analysis. Before the analysis, the samples were filtered using 0.45 µm Millex-HN syringe filters with a nylon membrane. For the qualitative and quantitative determination of ATZ in the samples, a high-performance liquid chromatography system was used. The system used had an automatic injector (Sil-10A), a quaternary pump (CTO-10A), and a UV/Vis detector (SPD-10AV). The chromatographic separation was performed using a reversed phase Lichrosorb RP-18 column (250 mm×4.6 mm, particle size of 5 µm, 100 A, Phenomenex) and was carried out with isocratic elution with a mobile phase of acetonitrile/water (50:50, v/v) and flow rate of 0.6 mL/min. The detection was done at 220 nm, and the run time was 15 min. Qualitative and quantitative determinations were carried out using an external standard. The analytical curves were constructed using an ATZ standard (Chem Service, 98.1% purity) diluted in Milli-Q^®^ water. When necessary, the samples were diluted by a factor to fit the working range of the standard curves.

### Acute toxicity assay of ATZ in Pacu fingerlings

The fish were exposed to the LC_50_ for 96 h (28.58 mg/L), data previously obtained by Peterlini [32] in a static system. After acclimatization in the laboratory, the fingerlings were measured (mean total weight of 6.32±0.41 g and mean total length of 7.05±0.05 cm) and randomly distributed in four glass tanks (50 L) containing dechlorinated water. Four glass tanks were for the control group, and four were for the ATZ-exposed group (n=4 per glass tank), given a total number of 16 animals tested per group.

During experimentation period, the water parameters were evaluated. For the control group, the parameters were water pH 8.13±0.16, temperature 21.62±0.09°C, dissolved oxygen 8.61±0.46 mg/L, ammonia concentration 0.017±0.005 ppm, and hardness bland. For the ATZ-exposed group, the parameters were water pH 8.13±0.05, temperature 21.89±0.29°C, dissolved oxygen 8.61±0.46 mg/L, ammonia concentration 0.018±0.002 ppm, and hardness bland.

After completing 96 h of exposure, nine fish survived in treated group, so the same numbers of fish were used from control group. The animals were sacrificed by deep anesthesia in a glass tank with 2-phenoxyethanol (diluted 1:600 in dechlorinated water). Thus, nine fish were used for the preparation of blood smears, and six were used to collect tissue fragments.

All experimental protocols used in this work were approved by the Ethics Committee on Animal use of the University of Campinas (UNICAMP) under protocol #2378-1/A.

### Histopathological analysis by light microscopy

Representative samples from liver and kidney (trunk kidney) from each fish were fixed in Bouin’s solution at room temperature (RT) for 24 h and were washed with tap water for 12 h. The samples were subsequently processed for inclusion in Paraffin embedment. Sections (4 µm thick) obtained with an electronic microtome (Leica RM 2145) were stained with hematoxylin and eosin.

The stained sections were examined with a Nikon Eclipse E-800 light microscope (Nikon, Japan) equipped with a CoolSNAP-Pro color video camera (Media Cybernetics, San Diego, CA, USA), and images of the sections were captured using the Image Pro-Plus software (Media Cybernetics – version 4.1.1.2).

The degree of histopathological alterations in the sections of liver and kidney samples from each fish was evaluated semiquantitatively using the protocol described by Bernet *et al*. [33], by blind histological analysis performed by two experienced observers. Score values ranging from 1 to 3 were given according to the importance represented by the histological lesion in terms of hepatic and renal functionality. The attribution of these scores was done by classifying the observed alterations according to the criteria described by Bernet *et al*. [33] and Costa *et al*. [34]. The alterations and their corresponding score values are given in Table-1 [33,34].

**Table-1:** Histopathological alterations and respective scores assessed in the liver and kidney of *Piaractus mesopotamicus* exposed to atrazine.

Organ	Score	Alteration
Liver	1	Vacuolated cytoplasm^a^
	2	Vacuolated nuclei^a^
		Intracellular eosinophilic bodies^b^
	3	Focal necrosis^a^
Kidney	1	Intercellular edema^a^
	2	Intracellular eosinophilic bodies^b^
		Degeneration of cytoplasm^a^

a Bernet *et al*. [33];

b Costa *et al*. [34]

For the calculation of the degree of hepatic and renal damage, for each animal, a modification of the method of Poleksic and Mitrovic-Tutundzic [35] was employed. The alteration severity index (I), considering the score value (1, 2, or 3) given to each alteration as described above, was calculated according to the equation: I=Σ1+10 Σ2+100 Σ3. Once the index value (I) was obtained the severity of the impairment of the organ was classified as follows: I of 0-10=normal functioning; I of 11-20=light to moderate damage; I of 21-50=moderate to severe alterations; and I>100=irreparable damage [35].

### Histopathological analysis by transmission electron microscopy

Liver and kidney samples were fixed in modified Karnovsky solution (2.0% paraformaldehyde and 2.5% glutaraldehyde in 0.1 M phosphate buffer, pH 7.4) at 4°C for 24 h, which was followed by post-fixation with 1% osmium for 1 h at 4°C. After the washing and dehydration processes, the material was embedded in EMbed 812 epoxy resin. Ultrathin sections (60-70 nm) were contrasted with uranyl acetate and 2% lead citrate and examined with a Zeiss LEO–906E transmission electron microscope.

### Western blotting analysis of Hsp70 tissue content in liver

To detect the tissue content of Hsp70 in liver from both experimental groups, we have used a standard protocol of immunoblotting described previously [36]. Briefly, liver fragments were homogenized by sonication in an antiprotease cocktail buffer aliquots of liver homogenate (50 µg of total protein) were fractionated by electrophoresis using 10% polyacrylamide gels, and the proteins were then electrotransferred to nitrocellulose membranes (Bio-Rad). After staining with Ponceau S solution (Sigma) to check the efficiency of transfer, the membranes were blocked with 5% dry skimmed milk in Tris-buffered saline (0.01M) containing 0.05% Tween 20 (TTBS) for 4 h at RT, incubated overnight at 4°C with the anti-HSP70 antibody (dilution 1:1000; Sigma, cat number H 5147), followed by a 2-h incubation at RT with the specific secondary antibody conjugated with horseradish peroxidase in TTBS with 1% dry skimmed milk (dilution 1:1500; Sigma). After washing, the protein bands on the membranes were revealed using an enhanced chemiluminescence kit (SuperSignal West Pico Chemiluminescent Substrate, Pierce, Rockford, USA). Band intensities were quantified by optical densitometry using ImageJ analysis software (http://rsbweb.nih.gov/ij/). After stripping, the membranes used for Hsp70 detection were reblotted with an anti-β-actin antibody (dilution 1:300; Sigma, cat number A2228) used as a loading control. The values were expressed as a ratio of the Hsp70/β-actin signals.

### MN test in erythrocytes

**Figure-1:**
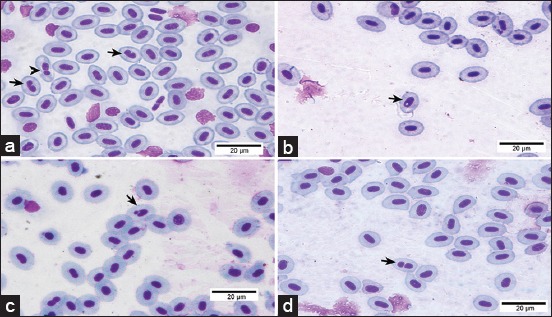
Micronuclei and nuclear abnormalities found in erythrocytes of Piaractus mesopotamicus from the control and atrazine-exposed groups. (a) Note the presence of lobed nuclei (arrowhead) and notched nuclei (arrow). (b) Note vacuolated nuclei (arrow). (c) Note micronuclei (arrow). (e) Note binuclear cell (arrow). 10% Giemsa solution.

Peripheral blood samples were taken from the caudal vein of the fish into heparinized microhematocrit capillary tubes by dissecting the caudal fins. Peripheral blood smears were air-dried overnight, and after fixation in pure methanol for 10 min, they were stained with 10% Giemsa solution for 10 min. Five slides were prepared for each fish, and 2,000 cells were double-blind scored from each slide. Using a light microscope, the number of MN and nuclear abnormalities (NAs), such as blebbed nuclei, lobed nuclei, vacuolated nuclei, and notched nuclei, was registered in each Pacu (Figure-1) following the morphological criteria described by Carrasco *et al*. [37], and binuclear cells were also counted [38]. The data of nuclear alterations were expressed as the total NA calculated by the sum of all NA.

### Statistical analysis

Comparative analyses of the alteration severity index (I) of the kidney and liver were performed using the Chi-square test, and the significance level was set at p<0.0001. The mean frequencies (±standard deviation) of MN and NAs of the treated group were compared with those of the control group using the Mann–Whitney U-test. Student’s t-test was used for comparing the two experimental groups in the case of the immunoblotting data (expressed as means ± standard error of the mean). The significance level used for the past two comparative analyses was set at p<0.05. All statistical analyses were performed using the GraphPad Prism Software version 5.00 (GraphPad Software, San Diego, CA, USA).

## Results

### Mortality

No mortality occurred in the control group during the acute assay. In the ATZ group, seven fish died out of a total of 16 initially put in experimentation.

### Quantitative evaluation of ATZ concentration in the acute assay

No trace of ATZ was detected in water samples from tanks destined to the control group. In water samples from the aquaria of the treatment group in the static system, where the nominal concentration of ATZ applied was 28.58 mg/L, the real concentrations detected at different periods after the addition of the test solutions were as follows: 1 h=24.68±1.06 mg/L, 24 h=23.56±0.19 mg/L, and 96 h=12.08±1.87 mg/L.

### Histopathological findings

#### Light microscopy

**Figure-2:**
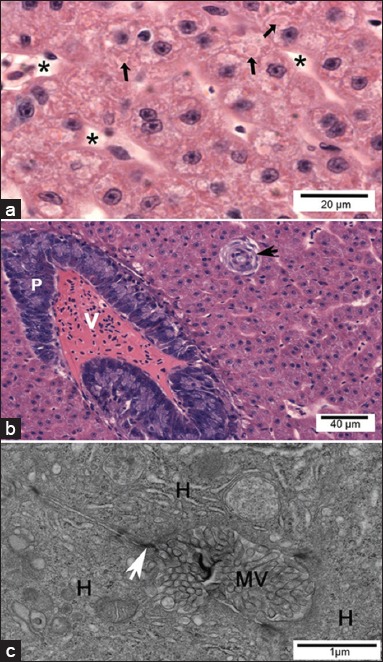
Light micrographs and transmission electron micrographs of control Pacu liver. (a) Liver parenchyma consisted mainly of hepatocytes presenting the cytoplasm voluminous with little vacuolization. Note the presence of cytoplasmic hyaline inclusions (arrows). Sinusoids are also observed (*). (b) Note the intrahepatic exocrine pancreas (P), vein (V), bile duct (arrow). (a and b) H and E. (c) Bile canaliculus formed by the plasma membranes of hepatocytes (H), where junctional complexes are noted between neighboring cells (white arrow) and the canaliculus lumen filled with hepatocyte microvillus processes).

In control Pacu fingerlings, the liver parenchyma consisted mainly of hepatocytes, bile ductules and ducts, and sinusoidal capillaries. The hepatocytes presented as large cells with little cytoplasmic vacuolization, a centrally located round nucleus containing noncondensed chromatin and a prominent nucleolus (Figure-2a). The presence of small eosinophilic cytoplasmic inclusions was rarely observed in these cells (Figure-2a). Exocrine pancreas with intrahepatic location, associated with the adventitia of branches of the portal vein, was also characteristic of the Pacu liver (Figure-2b).

**Figure-3:**
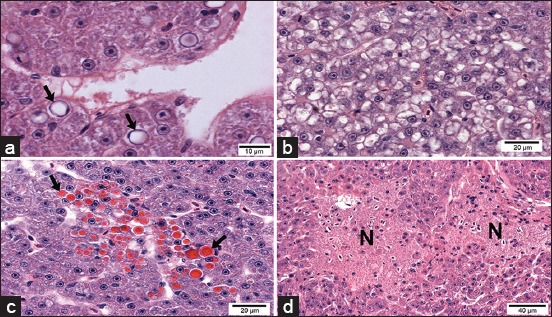
Light micrographs of the liver from Pacu fingerlings exposed to 28.58 mg/L of atrazine. (a) Hepatocytes are showing nuclear vacuolization (black arrows). (b) Hepatocytes are showing cytoplasmic vacuolization. (c) The liver is showing hepatocytes with hyaline inclusions (black arrows). (d) Necrotic areas (N) (H and E).

In fingerlings exposed to ATZ, histopathological changes were observed in hepatocytes, such as nuclear vacuolization (Figure-3a), cytoplasmic vacuolization (Figure-3b), cytoplasmic hyaline inclusions (HIs) (Figure-3c), and focal necrosis (Figure-3d). The HIs were circular, homogeneous in appearance and showed strong eosinophilic staining. The size and quantity of these HIs varied among hepatocytes; in some cases, the inclusion was sufficiently large to occupy the entire cytoplasm or occurred as several small inclusions that partially occupied the cytoplasm. Hepatocytes with HIs were arranged in foci (Figure-3c) or distributed throughout the parenchyma, with the latter arrangement being more common. Histopathological analysis under light microscopy revealed no alterations in the bile duct system (data not shown).

**Figure-4:**
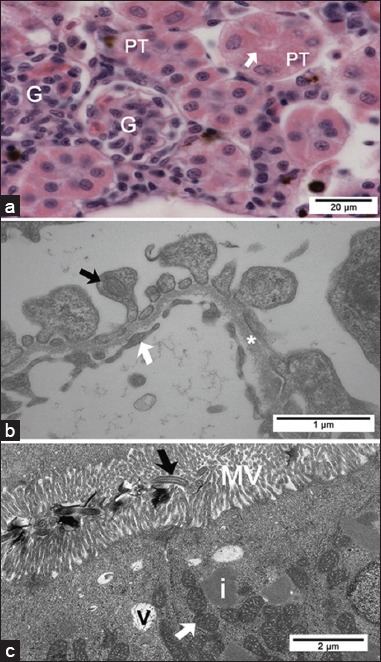
Light and transmission electron micrographs of control Pacu kidney. (a) Note the glomerulus (G) and proximal tubules (PT) with brush border (arrow) (H and E). (b) Pedicels of podocytes (black arrow), basal lamina (*) and the endothelium (white arrow) of the glomerulus can be seen. (c) Note in the proximal tubule: Microvilli (MV) and cilia (black arrow), electron lucent vesicles (v) in the apical region, inclusions with different electron density (i), and mitochondria (white arrow).

Control Pacu fingerlings showed the typical trunk kidney morphology of teleosts with glomerular kidneys. The kidney consisted of nephrons formed by a glomerulus and renal tubules (Figure-4a), which were surrounded by hematopoietic tissue.

**Figure-5:**
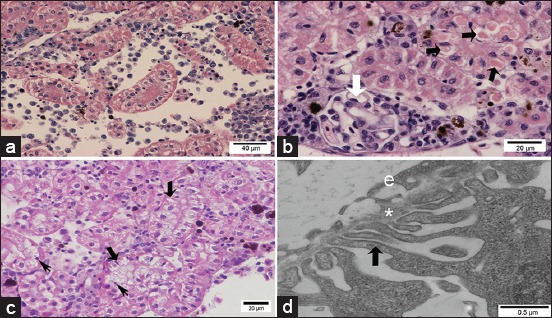
Light and transmission electron micrographs of Pacu kidney from the group treated with 28.58 mg/L atrazine. (a) Area with edema and proximal tubules (PT) with vacuolated cells. (b) Proximal tubules are showing cytoplasmic hyaline inclusions (black arrows). Note the normal structure of the glomerulus (white arrow). (c) Note tubular degeneration in PT with vacuolated cells (thick arrows) and picnotic nucleus (thin arrows). (d) Glomerulus is showing podocyte pedicels (black arrow), basal lamina (*), and the endothelium of the glomerulus (e) without alterations. (a-c) H and E.

In the kidney of ATZ-treated fish, areas with edema (Figure-5a) and tubular morphological changes indicative of tubular degeneration were observed only in the proximal tubules (PT); these alterations included cytoplasmic vacuolization, picnotic nucleus (Figure-5a and c), and HIs (Figure-5b) that showed similar appearance to those HI observed in the liver.

No morphological alterations were seen using light microscopy in glomerulus from ATZ-treated fish (Figure-5b).

The frequency of the index of severity impairment (I) of the kidney and liver for each fish from the control and treated groups is presented in Table-2, and the comparative analysis indicated a significant difference among the groups (p<0.0001). Overall, the Pacu liver displayed a relatively higher sensitivity to ATZ-induced damage as compared to the kidney (Table-2).

**Table-2:** Frequency of *Piaractus mesopotamicus* with each liver and kidney alteration severity index.

ATZ concentrations	Index of severity of impairment (frequency %)			

	0-10 Functionally normal organ	11-20 Slight to moderate damage	21-50 Moderate to heavy damage	>100 Irreparable damage
Liver				
Control (0.0 mg/L)	100***	0	0	0
Treated (28.58 mg/L)	0	0	33.33***	66.66***
Kidney				
Control (0.0 mg/L)	100***	0	0	0
Treated (28.58 mg/L)	0	50***	50***	0

*** p<0.0001 (Chi-square test),

n=6 per group, ATZ: Atrazine

#### Transmission electron microscopy

**Figure-6:**
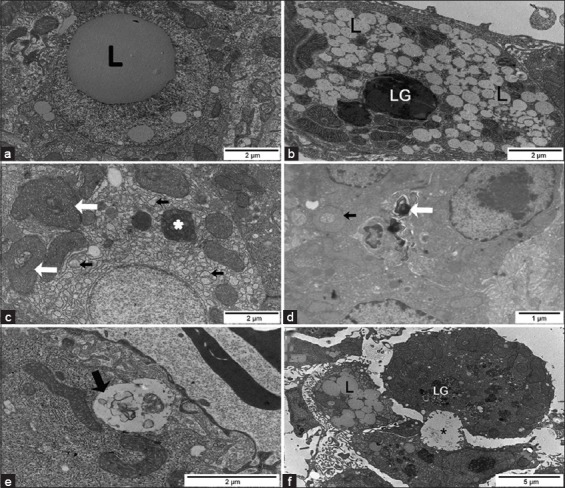
Transmission electron micrographs of the liver from Pacu treated with 28.58 mg/L of atrazine. (a) Hepatocyte with a nuclear lipid inclusion (L). (b) Hepatocyte with several cytoplasmic lipid inclusions (L) and a lipofuscin granule (LG). (c) Hepatocyte with mitochondria displaying altered morphology (white arrows), swollen rough endoplasmic reticulum (black arrows), and an autophagosome (*). (d) Hepatocytes with nuclear margination of the chromatin and myelin figure in the bile canaliculus lumen (white arrow). Note mitochondria displaying altered morphology (black arrow). (e) Note blebs of hepatocytes (black arrow) within the perisinusoidal space. (f) Degenerated hepatocytes with a large intercellular space, L and LG, and the bile canaliculus (*) shows dilatation with few and short microvilli.

The control group had hepatocytes and the biliary system with a typical ultrastructure of this species (Figure-2c). In fingerlings exposed to ATZ, the nuclei of most hepatocytes contained lipid inclusions (Figure-6a) or presented nuclear margination of chromatin (Figure-6d). The ultrastructure of the hepatocyte cytoplasm presented several alterations, including a large number of lipid inclusions (Figure-6b), a high amount of lipofuscin granules (LG) (Figure-6b and f), degranulation and dilation of the rough endoplasmic reticulum (Figure-6c), swollen mitochondria with an occasional loss of cristae, mitochondria with altered shape, and a large amount of lysosomes and autophagosomes (Figure-6c-e). Degenerated hepatocytes with a large intercellular space and bleb formation were also observed (Figure-6e and f). The bile canaliculi showed a partial loss of microvilli and sometimes myelin figures in the lumen (Figure-6d and f). There were no cellular alterations in bile preductular epithelial cells and other structures of the biliary system (data not shown).

**Figure-7:**
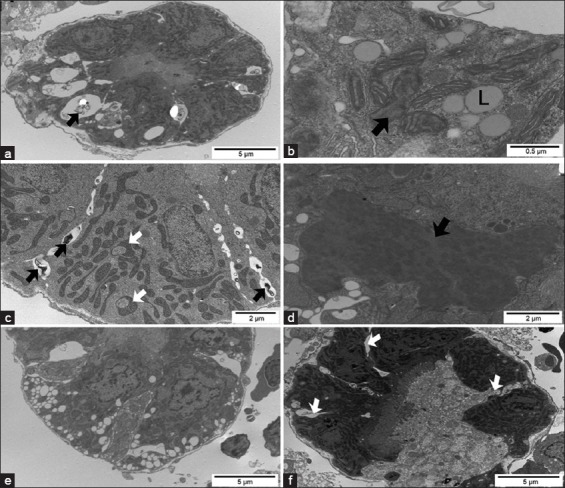
Transmission electron micrographs of the kidney from Pacu treated with 28.58 mg/L of atrazine. (a) Proximal tubule (PT) cells with a large number of electron lucent vacuoles containing myelin figures (black arrow) in the basal region of the cytoplasm. (b) PT with alterations in the organization of mitochondrial cristae (black arrow) and cytoplasmic lipid inclusions (L). (c) PT with noted mitochondria with altered shape (white arrow) and enlarged intercellular space containing myelin figures (black arrow). (d) Heterogeneous inclusion (black arrow) in cells of the proximal tubule. (e) Cells of the PT showing cytoplasmic vacuolization, nuclear chromatin margination, and enlarged intercellular spaces. (f) Degenerated proximal tubule showing relatively large intercellular spaces (white arrows) in a less affected area.

Fingerlings from the control group showed the typical structure of glomerulus and podocytes (Figure-4b). The renal tubules showed a regular arrangement, and the epithelial cells from PT had a well-developed brush border and some cilia (Figure-4c). In the kidneys of ATZ-treated fish, no alterations were noted in glomerulus (Figure-5d), but the PT presented several changes. The basal region of PT cells showed a large number of electron lucent vacuoles that occasionally displayed inside myelin figures or flocculent material (Figure-7a and e), dilated intercellular spaces containing myelin figures (Figure-7c and f), lipid inclusions (Figure-7b), alterations in the organization of mitochondrial cristae and in the mitochondrial shape (Figure-7b and c), and heterogeneous cytoplasmic inclusions (Figure-7d). The extent of PT cellular degeneration ranged from moderate, which included cells with alterations of the nuclear shape, chromatin margination, enlarged intercellular space, vacuolated basal region (Figure-7e and f), to severe, which included cells with a total loss of cellular structure and organization (Figure-7f).

### Expression of Hsp70 in liver

**Figure-8:**
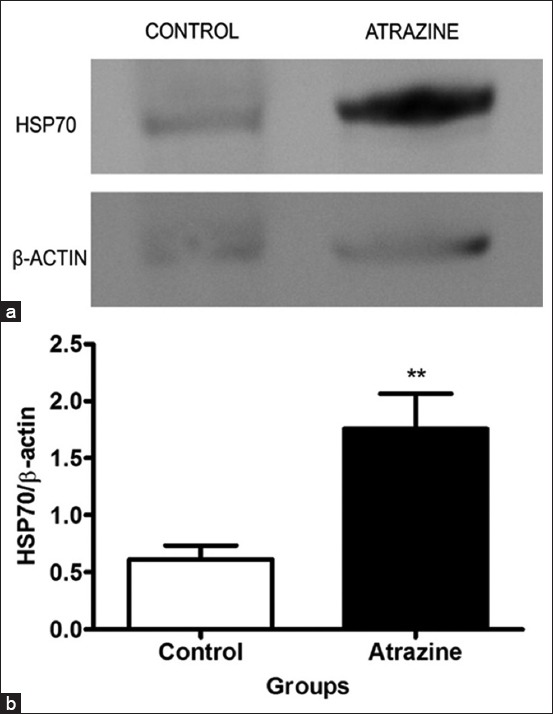
Pacu exposed to atrazine (ATZ) (28.58 mg/L) for 96 h. (a) Lysates of the liver were immunoblotted for Hsp70, and β-actin was used as a loading control. An acute treatment with ATZ induced a significant increase in the liver content of Hsp70 in Pacu fish. (b) In the graph, it is noted that ATZ induced a significant increase in the liver content of Hsp70 compared to the control group. The bars represent the means ± standard error of the mean of 10 membranes from five animals group. **p<0.01 (Student’s t-test).

As shown in Figure-8a and b, the 96-h treatment with ATZ (28.58 mg/L) induced a significant 2.8-fold increase in the liver content of Hsp70 in Pacu fish compared to the control group.

### MN test in Pacu erythrocytes

Pacu fingerlings exposed to 96 h of treatment with ATZ at its LC_50_ (28.58 mg/L) did not show significant differences in the frequency of MN and NAs in erythrocytes (p<0.05) when compared with the control group although the treated group presented a subtle increase in these frequencies, as shown in Table-3.

**Table-3:** Mean frequency±standard deviation (%) and total cell scored of MNs and NAs observed in erythrocytes of *Piaractus mesopotamicus* in atrazine acute assay.

ATZ concentrations	MN (%)	MN total	NA (%)	NA total
0.0 mg/L	0.01±0.01	9	1.49±0.68	1349
28.58 mg/L	0.014±0.01	13	1.89±0.74	1706

n=9 per concentration, total number of cells screened/group=90,000. No significant difference by Mann–Whitney U-test with p<0.05. MN=Micronucleus, NA=Nuclear abnormalities, ATZ: Atrazine

## Discussion

The environmental impact of agrochemicals and other xenobiotics is a worldwide concern. In Brazil, the last major environmental accident with an agrochemical involved a spill of approximately 8,000 L of the organochlorine endosulfan in water resources that occurred in 2008 in Rio de Janeiro State [39]. Recently, the dam break of the Samarco in Minas Gerais, widely reported by the media, resulted in a release of large amounts of heavy metals in the Rio Doce River with extremely negative consequences for the environment and the local population. Considering the increasing and indiscriminate use of herbicides, such as ATZ and the fact that high concentrations of this herbicide have already been detected in Brazilian rivers [11], studies on the acute effects of high concentrations of herbicides in Brazilian icthyofauna are important and can certainly yield significant information to guide the establishment of standards and conduct in case of an environmental accident involving water resources. Indeed, studies in line with this idea have been conducted by researchers from emerging national economies, where an increasingly use of ATZ in agriculture has been reported [38]. The present study reports the acute effects and sensitivity of a Brazilian fish to ATZ at experimental conditions and can constitute the base for further works employing sublethal and realistic concentrations of this xenobiotic.

The exposure of Pacu fingerlings (6.32±0.41 g of body weight) to the LC_50_ (28.58 mg/L) of commercial ATZ, determined previously [32], indicated a relatively high sensitivity of the Pacu in comparison with trout, which is considered a very xenobiotic-sensitive teleost species, since the determined 96-h LC_50_ of ATZ was 38.0 mg/L for trout fingerlings weighting 1.3 g [40]. In fingerlings of *Rutilus frisii kutum* (2.6 g), a marine teleost, the 96-h LC_50_ for ATZ was 24.94 mg/L [41], which was close to that seen in Pacu fingerlings but with higher body weight. The histopathological changes observed in Pacu exposed to ATZ are in accordance with findings described in the previous studies with this herbicide in the liver [17,42] and kidney [16,18] of other teleosts.

In relation to the small eosinophilic cytoplasmic inclusions rarely observed in liver from control Pacu, they may represent, according to Wolf *et al*. [43], a variety of different cellular and extracellular substances, including protein droplets, lipofuscin, ceroid, phagocytosed erythrocytes, iron pigments, and/or apoptotic hepatocytes. When present at low numbers in the liver of clinically healthy fish, as seen in our study, such inclusions do not necessarily indicate a pathological degree of liver degeneration. However, in contrast with the control group, the HI, characterized by strong eosinophilia, were the most frequently observed cellular alteration in the liver and kidney of Pacu acutely exposed to ATZ. HI has not been previously reported in fish liver after acute exposure to ATZ or to other xenobiotics. However, inclusions in the kidney of rainbow trout, similar to that described here in Pacu, were seen after acute toxicity with Metribuzin, a pesticide from the triazine family [44]. These inclusions were described as hyaline degeneration by the authors, but their presence was not correlated with any specific damage to the renal tubules [44].

Although HIs have not been reported in fish liver after acute toxicity tests, chronic toxicity, and biomonitoring studies have shown similar hepatic inclusions. Marchand *et al*. [45] observed cytoplasmic eosinophilic granules in the hepatocytes of fish collected from contaminated water systems, but they did not establish a clear relationship between this finding and any specific physiological disturbance. Costa *et al*. [34], who evaluated the estuarine ecological risk by assessing the hepatic histopathological appearance in the laboratory and *in situ* tested fish, described that these inclusions contained protein and a protein-bound lipid and suggested that they could be enlarged lysosomes. In fact, several authors have documented that ATZ exposure increases the number and size of LG and induces an enlargement of the secondary lysosomes in hepatocytes [42] and renal tubular cells [16,21]. Strmac and Braunbeck [46], who observed an increase in the number of lysosomes in hepatocytes of rainbow trout (*Oncorhynchus mykiss*) exposed to a mixture of 20 pollutants, suggested that the proliferation of lysosomes might represent a general adaptive mechanism to compensate for an increased turnover rate of cellular components during oxidative stress. Therefore, our ultrastructural findings showing the increased number of lysosomes and enlarged LG in hepatocytes as well as the presence of large heterogeneous inclusions in PT cells in the kidney after ATZ treatment may correspond to the HIs seen by light microscopy in these cells.

The cytoplasmic vacuoles observed within the PT cells of Pacu treated with ATZ were also described by other authors evaluating the effects of ATZ in fish kidney [20]. Taking into consideration the possible lysosomal impairment induced by ATZ, the ultrastructural aspect of these vacuoles is similar to that described in the drug-induced phospholipidosis, characterized by unicentric or multicentric lamellar bodies encapsulated by membrane and myelin figures [47].

We observed that the ATZ-induced alterations in the kidney occurred specifically in the PT, as commented above. However, few studies that have examined the effect of chemicals in fish kidney identified the specific region of the nephron that was most affected by the contaminants. Oulmi *et al*. [16], who examined the selectivity of renal tubule damage in rainbow trout exposed to sublethal concentrations of ATZ, noted that the PT were more sensitive to ATZ than distal tubule, which is consistent with the results described herein in Pacu. In mammals, the PT is the main target for several xenobiotics [48,49]. However, in fish, studies examining the specific location of the enzymes involved in renal detoxification are scarce. Zodrow *et al*. [50] showed that acute exposure to 2,3,7,8-tetrachlorodibenzo-p-dioxin induced an increase in the expression of cytochrome P450 1A in renal PT of zebrafish, as revealed by immunohistochemistry, suggesting that this nephron segment may play a role in the body detoxification process.

The results of the semiquantitative histopathological analysis performed in the liver and kidney of Pacu exposed to ATZ revealed functional impairment of both organs. However, the liver seemed to be a more sensitive histological biomarker because around 67% of the animals presented areas of focal necrosis resulting in a high index of liver impairment, considered as irreparable damage, while 50% of the treated animals displayed moderate to marked changes in the kidney. This result reinforces the importance of the application of a semiquantitative methodology to evaluate the actual degree of severity induced by a xenobiotic in every analyzed target organ. This strategy has been extensively applied in studies of toxicity induced by xenobiotics in teleost [34,45,51-53], but to the best of our knowledge, it has not been used to investigate the effects of ATZ in liver and kidney of fish.

Regarding the alterations to the cell membranes and mitochondria (i.e., swollen mitochondria with loss of mitochondrial cristae) observed in the liver and kidney of Pacu after ATZ treatment, they are commonly related to oxidative stress and peroxidation of lipids [54]. ATZ has been shown to induce oxidative stress in the liver [17,25] and kidney [18] of teleosts. Lipid peroxidation, which can alter the physicochemical properties of membrane lipid bilayers, resulting in severe cellular dysfunction [55], is one of the molecular mechanisms involved in pesticide-induced toxicity [56]. In addition, it is well known that the increased generation of reactive oxygen species is one of the factors that can reduce the cellular antioxidant capacity, which in turn results in oxidative stress [57]. Interestingly, Liu *et al*. [58] demonstrated that ATZ induced a dramatic and sustained elevation of O_2_ and H_2_O_2_ over the course of the *in vitro* toxicity assay in grass carp cell line. The morphological changes in mitochondria have been also linked to an impairment of their energy production capacity, as reviewed by Benard and Rossignol [59]. Padmini and Rani [60], studying the liver of *Mugil cephalus* from an uncontaminated and a contaminated estuary zones, in the East Coast of India, observed morphological alterations in hepatocyte mitochondria of fish from the contaminated zone, which were associated with a significant decrease in cellular adenosine triphosphate/adenosine diphosphate ratio. In accordance, it has been demonstrated that xenobiotics, including ATZ [58], can induce alterations in bioenergetics [61,62].

In addition, we observed that the LC_50_ of ATZ induced a significant increase in the expression of Hsp70 in Pacu liver, which is consistent with the morphological findings described above, suggestive of the occurrence of oxidative stress in hepatocytes. Hsps, particularly the Hsp70 family, are known to mediate cellular protection against oxidative stress [63]. In fact, the expression of Hsp70, which is a highly conserved protein family in vertebrates, has been suggested as an adequate biomarker of oxidative stress induced by xenobiotics in teleosts [27,28,64], which is reinforced by our data.

Regarding the genotoxic effects of ATZ, the International Agency for Research on Cancer considers ATZ as noncarcinogenic to humans although there is evidence of its carcinogenic action in experimental animals (International Agency for Research on Cancer - IARC, 2014) [31,38,65]. In this work, despite a tendency of higher frequencies of MN and NAs in the ATZ-exposed group, no significant difference in these frequencies was seen between the treated and control groups, considering 90,000 erythrocytes screened per group. In contrast, Nwani *et al*. [38], evaluating different concentrations of ATZ in *Channa punctatus* (10.6, 21.2 and 31.8 mg/L) in a semi-static system, have reported a significant increase in the MN frequencies in erythrocytes only at the highest concentration tested of ATZ after 96 h (but not 24 h) of exposure. Cavas *et al*. [31] also described a significant increase in the frequency of MNs and NAs in *Carassius auratus* exposed for 6 days to different concentrations of a commercial formulation of ATZ, but not after pure ATZ. Although the reason for the data discrepancy among those studies and our data are still unknown, it may reflect differences in sensitivity among fish species to different concentrations, formulations, and exposure conditions of ATZ. In line with this idea, Sato *et al*. [66], studying the induction of MN by six different clastogenic substances in three different mouse strains, have shown significant differences in the susceptibility among these strains. In addition, differences in the formulation of commercial ATZ have also been pointed out as a factor that may explain the differences in the genotoxic effects of this herbicide [31]; in this respect, Nwani *et al*. [38], that observed acute genotoxic effect in fish, used ATZ from a different source (Rasayanzine manufactured by Krishi Rasayan Exports, Kolkata, India) than us (Gesaprim 500 from CIBA-GEIGY, Syngenta).

## Conclusion

Based on the results presented, we can suggest that Pacu fingerlings may be a good bioindicator for testing freshwater contamination because they showed sensitivity to ATZ when compared to other species of teleost. Our findings revealed that both liver and kidney are adequate biomarkers to evaluate the acute toxicity induced in Pacu by ATZ. We found that the liver is a more sensitive target organ, as indicated by the semiquantitative histopathological analysis. This observation emphasizes the importance of the use of semiquantitative methods and not only qualitative analysis to evaluate the severity of organ damage in xenobiotic-induced toxicity tests. Morphological and molecular analysis indicated that ATZ induces cellular stress in the liver and kidney, although no significant differences in the frequency of MN and NAs were observed between control and treated Pacu.

## Authors’ Contributions

SA designed the experiment. SA and CBC supervised the experiment and participated in the analysis and discussion of the data. PPP participated in the experimental work, analysis, and discussion of the data. MCD and VAM collaborated with the experimental work. SCNQ performed the water analysis to evaluate the actual concentration of atrazine and collaborate in writing this part of the manuscript. All authors read and approved the final manuscript.
